# Exploring Existential Loneliness Among Frail Older People as a Basis for an Intervention: Protocol for the Development Phase of the LONE Study

**DOI:** 10.2196/13607

**Published:** 2019-08-14

**Authors:** Anna-Karin Edberg, Ingrid Bolmsjö

**Affiliations:** 1 The Research Platform for Collaboration for Health Faculty of Health Sciences Kristianstad University Kristianstad Sweden; 2 Faculty of Health & Society Malmö University Malmö Sweden

**Keywords:** study protocol, loneliness, existential, frail older adults, qualitative research, health personnel

## Abstract

**Background:**

International research concerning end-of-life issues emphasizes the importance of health care professionals (HCPs) being prepared to deal with existential aspects, like loneliness, in order to provide adequate care. The last phase of life is often related to losses of different kinds, which might trigger feelings of isolation in general and existential loneliness (EL) in particular. There is a large body of research concerning loneliness among older people in general, but little is known about the phenomenon and concept of EL in old age.

**Objective:**

This study aims to describe the framing, design, and first results of the exploratory phase of an intervention study focusing on EL among older people: the LONE study. This stage of the study corresponds to the development phase, according to the Medical Research Council framework for designing complex interventions.

**Methods:**

The LONE study contains both theoretical and empirical studies concerning: (1) identifying the evidence base; (2) identifying and developing theory through individual and focus group interviews with frail older people, significant others, and HCPs; and (3) modeling process and outcomes for the intervention. This project involves sensitive issues that must be carefully reviewed. The topic in itself concerns a sensitive matter and the study group is vulnerable, therefore, an ethical consciousness will be applied throughout the project.

**Results:**

The results so far show that EL means being disconnected from life and implies a feeling of being fundamentally separated from others and the world, whether or not one has family, friends, or other close acquaintances. Although significant others highlighted things such as lack of activities, not participating in a social environment, and giving up on life as aspects of EL, the older people themselves highlighted a sense of meaningless waiting, a longing for a deeper connectedness, and restricted freedom as their origins of EL. The views of HCPs on the origin of EL, the place of care, and their own role differed between contexts.

**Conclusions:**

The studies focusing on identifying the evidence base and developing theory are published. These results will now be used to identify potential intervention components, barriers, and enablers for the implementation of an intervention aimed at supporting HCPs in encountering EL among older people.

**International Registered Report Identifier (IRRID):**

RR1-10.2196/13607

## Introduction

### Background

Research conducted by our group shows that older people receiving municipal care during the last period of life [1], older people in general [2], and severely ill people at the end of their lives [3] all describe the importance of being able to talk to others about existential aspects of life, including their approaching death. Our previous research shows that one of the most significant challenges for health care professionals (HCPs) is communication about existential issues that occur at the end of life [4]. There is, thus, a need for more knowledge about how we can facilitate communication between frail older people and their significant others, and also improve the quality of care. This paper describes the framing, design, and first results of the first exploratory phase of a study focusing on existential loneliness (EL) among older people, the LONE study. This phase corresponds to the development phase, according to the Medical Research Council (MRC) framework [5], and contains both theoretical and empirical studies as a basis for the development of an intervention focusing on supporting HCPs when they encounter EL among older people.

### Rationale for the Study

International research concerning end-of-life concerns emphasizes the importance of HCPs being prepared to deal with existential issues in order to provide adequate care and services. One such central existential issue is loneliness. Loneliness is often related to either physical aspects of life, where a need for closeness and touch are in focus, or to social isolation, where a need for human relationships are in focus [6]. Both these aspects can appear at any time throughout life but are more often present when people get older and their social networks shrink. Another aspect of loneliness, described in the literature as EL or existential isolation, can also be experienced throughout life. EL concerns the concepts of meaning and hope [7,8], as well as the awareness of being vulnerable and mortal as a human being [9,10]. During times of grief, worry, and illness, feelings of EL might be enhanced. The last phase of life is often related to losses of different kinds, which might trigger feelings of loneliness in general, and EL in particular. There is a large body of research concerning loneliness among older people in general [11], but little is known about the phenomenon of EL in old age. We, therefore, aim to focus specifically on EL in this project.

### What Do We Already Know?

It is not possible to deal with aspects of EL without taking a philosophical point of departure. Existentialists such as Frankl [12], Buber [13], and Sartre [14] have all focused on questions of human existence and the meaning of life. The literature highlights key issues such as freedom, choice, responsibility, and anxiety. The fundamental idea is that we are all, as human beings, free both to choose and to not choose; we alone bear responsibility for all our choices, and as a consequence we face anxiety. In the scientific literature, EL has been looked at from different perspectives, such as philosophy, caring science, social science, gerontology, and psychiatry [7,9,15-18]. Several of these studies confirm that the existential component in loneliness often has an indirect relationship with the concept of meaning [7,8] as well as a search for meaning and hope in the present situation, thereby reducing suffering [19]. When it comes to nursing research, previous research has tended to relate EL to existential suffering, such as a fear of death and being alone [20], instead of considering it as a continuum that can be constructive if it is encountered and validated by others [21]. Other researchers have highlighted that EL is not primarily about suffering but should rather be understood as an awareness of being vulnerable and mortal [9,10]. The concept of EL, thus, has a broad empirical significance, as it is often reinforced when a human being is left on their own in a threatening situation, such as during severe illness [8]. Ettema et al [8] have emphasized that EL is a multifaceted concept that is far from clear, and theoretical as well as empirical research is needed.

EL is often described in relation to dying and death and especially concerning palliative care [22-24]. One of the most difficult challenges for health care staff is caring for people in palliative care at the end of life, as shown by discussions with the patients about their dying and death [24,25]. Similar results have also been reported in studies involving HCPs working in municipal care and services for older people; communicating about dying and death, as well as encountering older peoples’ existential needs, was found to be difficult and something that the staff tried to avoid [26]. This, in turn, affected the residents’ ability to speak about their existential needs.

HCPs working in palliative care have described how, although they are trained to take care of patients in different crises, they end up experiencing their own unwanted thoughts and feelings about death during that process. This results in a sense of vulnerability, and they thus felt less prepared to handle their patients’ existential suffering [27]. Many researchers have pointed out that HCPs must understand their own EL to handle the situation of a patient who is close to death [8,19]. In addition to difficulties in dealing with their own anxiety about death, staff also experienced difficulties in communicating with their patients about EL. Thus, the way the HCPs respond to questions concerning EL is affected by both their own view of dying and their own difficulties in communicating existential issues in encounters with patients or residents and their families.

The next of kin are another group that must be considered when investigating older people’s experiences of EL. Although patients may struggle with existential concerns at the end of life, these issues are seldom brought into focus in their care [28] or discussed with their families or close friends. An interview study with 17 participants found that the experience of being next of kin to an older person in the last phase of life was understood as being a devoted companion during the transition toward the inevitable end [29]. This meant that the next of kin were present regardless of whether they were caregivers or not, and that they shared aspects of the old person’s everyday life during this final transitionary phase as well as during their last moments. The experience of being next of kin further meant living in the shadow of death, focusing on the needs of the dying person, making adjustments to everyday life, feeling a major responsibility for this person, struggling with the health and social care system, and gaining strength from support [29]. As the next of kin are involved in the care and are emotionally affected by the situation, their need for support should also be acknowledged.

The way in which HCPs encounter patients’ existential issues is clearly related to the context of care. Studies in palliative care have shown that there is a need for research concerning existential issues related to different contexts and cultures of care, as well as the influence of context on the provision of care and service. This knowledge is needed to end patients’ sense of isolation and contribute to more health-promoting care [30-32]. We know that specialized and acute care are predominantly characterized by medical life-saving actions [33], while hospice care is characterized by efforts toward a healing environment, and an atmosphere that affirms life and promotes a good death [34]. In special accommodations for older people, there is a strong focus on activities to promote independence [26] even though most people die within the first year after moving in [35]. Thus, the culture and discourse within different care contexts clearly affects the experience of EL among the people being cared for, as well as the HCPs’ possibilities to meet with and confirm the experiences of the patients and their next of kin.

There is a lack of any methods for supporting HCPs in identifying and handling EL. Several studies (eg, [24]) emphasize the need for research focusing on how, despite the presence of multiple diseases and severe symptoms, the end of life can be a positive phase of life where hope and joy can coexist with grief and suffering. However, other researchers [8,19] point to the need for clarity concerning both theoretical and empirical evidence on the phenomenon of EL, so that HCPs can better handle it in their daily work and see it from the patients’ perspective. If EL is identified and confirmed, both as suffering and a route to inner peace, this might support a feeling of deeper understanding and increased well-being for patients or residents and their significant others, as well as for HCPs.

### Aims

We know that caring for people at the end of life is existentially challenging, as dying and death are constantly present, especially in the care of older people. As all frail older people are by definition in the last phase of life, we can assume that they experience EL to some degree. However, EL is a multifaceted phenomenon that needs to be further explored. On the basis of earlier research, we also know that HCPs need support to encounter older people’s thoughts about life and death. The aims of the LONE study are thus to:

1. Identify the evidence base for EL, by investigating the aspects and dimensions of EL.
2. Develop a theoretical frame for an intervention by investigating:
  - The meaning of EL from the perspective of frail older people and how EL can be eased.
  - Significant others’ experiences of encountering EL, and how they handle situations when their relatives experience EL.
  - HCPs’ experiences of encountering older people with EL, the ways in which they identify and handle EL, and their own needs for support.
  - EL in relation to different care contexts.
3. Prepare the modeling process and outcomes for the intervention by:
  - Identifying potential intervention components, as well as barriers and enablers for the implementation of the intervention.
  - Developing an outline for the intervention.

## Methods

### Overview

This paper describes the exploratory phase of a study focusing on EL among older people, which in turn will form a basis for the development of a complex intervention to support HCPs who care for these people. According to the MRC [5], aspects that determine the complexity of an intervention include the number of components, the number of interactions between components, the number of organizational levels targeted by the intervention, and the number and variability of positive outcomes. The complexity of an intervention is also influenced by how flexible the intervention is. The MRC highlights that the chance of an intervention’s success increases if the intervention is adjusted to the actual situation and prerequisites, and also grounded on a solid theoretical, as well as empirical, base [5]. The MRC also points to the fact that different stakeholders need to be involved in the phases of development, testing, and evaluation [36]. In the planning of the LONE study, we were guided by the MRC framework.

According to the MRC [5], there are 4 key elements in the process of developing and evaluating a complex intervention: development, feasibility and piloting, evaluation, and implementation. The first exploratory phase described in this paper corresponds to the development phase, which according to the MRC [5] includes: (1) identifying the evidence base; (2) identifying and developing the theory; and (3) modeling the process and outcomes. We have so far completed the studies relating to identifying the evidence base as well as identifying and developing the theory and are now (in June 2019) about to synthesize the results and model the process and outcomes.

### Identifying the Evidence Base

According to the MRC [5], the relevant existing evidence should be identified either through existing systematic reviews or by updating or conducting a new systematic review. As the literature concerning EL is multifaceted, it is very important to describe different aspects and dimensions (constructs) to know what we mean when we talk about EL. Building on an earlier systematic review and concept analysis of EL by Ettema et al [8], we conducted a review of the literature dealing with the concept and phenomenon of EL. The analysis focused on all relevant constructs for our understanding of EL and provided us with a definition, which, in a reciprocal process, has been used as a basis for analyzing the empirical material. This inclusive approach ensured that important perspectives in the studies were not lost.

### Identifying and Developing the Theory

According to the MRC, it is important to develop a theoretical understanding of the likely process of change by “drawing on the existing evidence or theory, supplemented if necessary by new primary research, for example interviews with those targeted by the intervention or involved in its development or delivery” [5].

**Figure 1:**
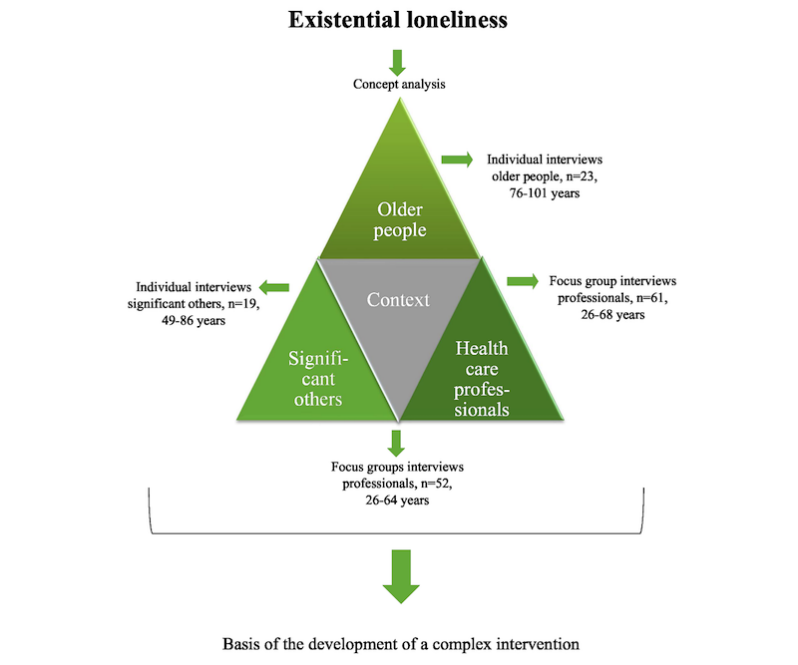
Overview of the areas included in the developmental phase of the intervention.

We, therefore, conducted empirical studies focusing on EL among older people through interviews with frail older people, their significant others, and HCPs. For an overview of the different parts, see Figure 1.

#### Frail Older Peoples’ Experiences of Existential Loneliness and How It Can Be Eased

In total, 23 patients and residents were interviewed concerning their experiences of EL. The criteria for inclusion were: being aged 75 years or older, being able to communicate, and being frail. Even though frailty has become an established concept in research, there is no agreed upon definition [37,38]. One way to operationalize frailty is through a quantitative summary of a number of frailty criteria. For example, Fried et al [39] recommend that 3 or more of the following 5 criteria should be used to identify and measure frailty in clinical practice: weakness, poor endurance, weight loss, low physical activity, and slow gait speed. Another study found that HCPs interpreted the concept somewhat differently, describing qualitative aspects such as being bodily weak and ill, lacking balance in everyday activities, and being dependent in everyday life [40]. As we had limited possibilities to investigate either quantitative or qualitative aspects of frailty when including the participants, we decided to use the somewhat more convenient criterion of being at least 75 years of age, and in need of long-term care or services. We argue that the indicator of long-term care and services fulfils as many as possible of the qualitative and quantitative descriptions of the concept, as in a Swedish context these descriptions are prerequisites for a decision about such support in municipal care [41], or for being registered in home health care or palliative care [42].

We included patients and residents from different contexts: primary care, home care, hospital, special accommodation, and palliative care. In primary care, home care, and special accommodation, the informants were identified by staff. At the hospital, the informants were identified by staff during their stay and invited to participate in connection with their discharge from hospital. In hospices, patients were identified and invited to participate after admission. Due to ethical aspects, people who were obviously in the very final stages of their life and receiving end-of-life care were not included.

The interviews with the patients and residents (n=23) were performed as individual narrative interviews focusing on the experience of EL. A total of 2 interviewers performed the interviews as a pair. The roles of the 2 interviewers were clear before the interview, as one performed the interview while the other sat to one side taking notes, and if needed, picked up clues that were missed during the interview. All interviews were performed by a doctoral student who was supported by a researcher with profound knowledge about interviews with older people and people in palliative care.

The interview started with an introduction to the concepts of loneliness and a deeper sense of loneliness; that is, EL. The informants were then asked for their thoughts about this and whether they could recall any situation when they felt that way. Probing questions were asked concerning the situation, their thoughts and feelings, whether they could or wanted to share their feelings with someone else, and in what way the experience of EL could be eased. At the end of the interview, the patients and residents were asked for permission to invite their next of kin for an interview. They themselves decided which significant other should be asked.

After the interview, the 2 interviewers reflected together on their experience of the interview and what they had learnt about EL during the interview. They also wrote a description concerning the informants’ situation and other contextual aspects. The interviews were recorded and transcribed. The first paper was analyzed with a phenomenological hermeneutical analysis [43], as we were interested in the meaning of EL, while the second paper aimed to describe experiences and was analyzed with conventional content analysis [44] using the contextual description as a frame.

#### The Perspectives of Significant Others

In all, 19 significant others were interviewed concerning their experience of encountering EL among their relatives. The informants were identified by the interviewed patients and residents (as described above), and were spouses, children, siblings, or other persons identified by the older person as being close to them. The interviews were performed as individual narrative interviews, with a focus on narrations about the experience of EL among their relatives. The majority of these interviews were performed by 2 researchers working as a pair, as described earlier. The interview started with an introduction to the concepts of loneliness and EL, and the informants were then asked if they could recall a situation when their relative expressed a feeling of a deeper sense of loneliness or EL. Follow-up questions were asked, such as “How did you notice?,” “Do you remember what you thought and felt?,” and “How did you handle the situation?”. The interviews were recorded, transcribed, and analyzed using conventional qualitative content analysis [44].

#### Health Care Professionals’ Experiences of Encountering Older People With Existential Loneliness, and Their Own Needs for Support 

In total, 11 focus group interviews with HCPs were performed. A focus group interview is based on the idea that people develop their views and narrations about their experiences in discussion with others [45]. Each focus group had a median of 6 participants from different professions (registered nurses, physicians, nurse assistants, social workers, occupational therapists, and physiotherapists), totaling 61 professionals. The focus groups were conducted in different care contexts: primary care, home care, prehospital care (ambulance), hospital, special accommodation, and palliative care. Each interview focused on the HCP’s experiences of encountering older people expressing EL. The interviews started with an introduction to the concepts of loneliness in general and EL in particular. The participants were then asked if they could recall a situation when, in their interpretation, a patient or resident experienced EL. Follow-up questions were asked, such as “How did you recognize the person’s experience?,” “How did you handle the situation?,” “What did it awake in you?,” “How are you prepared to handle such situations?,” and “What kind of support do you need?.” The interviews were recorded, transcribed, and analyzed using a combination of a deductive and an inductive approach, based on a theoretical framework by van Deurzen [46].

#### Existential Loneliness in Different Care Contexts

The interviews with HCPs from home care, hospital, special accommodation, and palliative care were analyzed with the material grouped according to context. The characteristics of each context were noted and compared, with a focus on the narratives of the HCPs. The material was analyzed using case study methodology [47].

### The Preparation of the Modeling Process and Outcomes

According to the MRC [5], modeling of a complex intervention before a full-scale evaluation can provide important information about the design of both the intervention and the evaluation. This phase will include preparation for this modeling through the identification of potential intervention components, barriers, and enablers for the implementation of the intervention through a qualitative evidence synthesis [48]. The aim of the synthesis will be to identify intervention mechanisms in the data already collected and analyzed in the previously described steps in the LONE study. The synthesis will further aim to identify, among other things, possible barriers and enablers for the intervention related to the context of care, and also search for possible explanations about how the intervention might work in different contexts.

We will also construct a preliminary outline of the intervention aimed at supporting HCPs in encountering EL, using concept mapping [49]. Concept mapping is a group-based method for developing a conceptual framework for an intervention or evaluation. The group participates in recurrent workshops with the aim of generating ideas and assessing their relevance. The results from these discussions are then structured in clusters, and their interrelation is illustrated visually using statistical methods. Different stakeholders (older people, significant others, HCPs, and health care managers) will participate in the concept mapping, and the qualitative evidence synthesis will be used as a basis for discussion and reflection. We have regularly consulted a review panel composed of different stakeholders (HCPs, significant others, and older people) throughout the study period. They will be especially important during the phase of actually developing the intervention.

### Ethical Aspects

This project involves several sensitive issues that need to be carefully reviewed. We are aware that the topic concerns a sensitive matter and that the study group is very vulnerable. We have, therefore, applied an ethical consciousness throughout the project. The voluntary nature of participation by patients, significant others, and HCPs has been emphasized in the information given out about the project and again before the interviews began. All participants gave their written informed consent to participate in the study. A contact person at each involved clinic, health care center, and special accommodation had the main responsibility of making the initial contact with the patients and residents that met the inclusion criteria so as to provide initial information about the project and ask if the researchers could contact them. The researchers then provided oral and written information about the study. After the interview, the contact person was responsible for observing whether the patient or resident had a need for a follow-up discussion. Other staff at the facility did not have any information about which patients or residents were included in the study. Following the interviews with the significant others, one of the interviewers contacted each participant by phone 2 days after the interview to ask whether they had any questions or thoughts that were awakened by the interview. Different pairs of interviewers performed the interviews with the patients or residents and the significant others to ensure that the interview with the significant other was not affected by knowledge about the patient or resident. This also prevented the risk of the significant other receiving information about what the patient or resident had said in their interview, as the researchers interviewing the significant other did not have this information.

### Ethics Approval and Consent to Participate

All participants gave their written informed consent to participate in the study. The study has been approved by the Regional Ethical Review Board in Lund, Sweden (ref: 2014-652).

### Availability of Data and Material

Due to the sensitive matter of the interviews and the fact that they are in Swedish, raw data will primarily not be made available for researchers outside the research group.

## Results

The studies aiming at identifying the evidence base and developing a theoretical frame for an intervention have so far resulted in 7 published papers from 2018 to 2019 and are summarized below.

### Identifying the Evidence Base

The concept analysis [50] focused on a clarification of what constitutes EL and describe its lived experiences. The aim was to provide a definition of EL that could function as a tool for identifying the phenomenon and for differentiating it from other kinds of loneliness. The definition that emerged was:

EL is the immediate awareness of being fundamentally separated from other people and from the universe, and typically, because of this awareness, experiencing negative feelings, that is, moods and emotions.

The crucial difference between subjective experience of being socially alone and EL appeared to be that social loneliness is about lacking intimate social relations, whereas EL is concerned with a more basic lack, namely, a feeling of being fundamentally separated from others and the world, whether or not one has a family, friends, or other close acquaintances. Thus, you might have close relations and not suffer from loneliness yet still experience EL [50].

### Identifying and Developing the Theory

#### Frail Older Peoples’ Experiences of Existential Loneliness and How It Can Be Eased

The 2 studies focusing on frail older people’s experience of EL [51,52] showed that EL mainly means being disconnected from life, that is, being trapped in a frail body, being met with indifference, having no one to share meaningful aspects of life with, and lacking meaning in life [51]. EL can, however, be eased when being acknowledged by others, that is, being the focus of others’ concern, encountering intimacy, and having meaningful exchanges of thoughts and feelings. EL could also be eased when bracketing negative thoughts and feelings, that is, when adjusting and accepting the present situation, viewing life in the rear‐view mirror, being in contact with spiritual dimensions, and being able to withdraw and distract themselves [52].

#### The Perspectives of Significant Others

The study focusing on significant others [53] showed that they interpreted that EL emerged when being increasingly limited in body and space, when being in the process of disconnecting, and when being disconnected from the outside world. As the significant others also discussed the reasons behind the experience of EL, we decided to pair and contrast these views with the older persons’ narratives [54]. The comparison showed that while significant others highlighted aspects of lack of activities, not participating in a social environment, and giving up on life, the older people themselves highlighted a sense of meaningless waiting, a longing for a deeper connectedness, and restricted freedom as origins of EL [54].

#### Health Care Professionals’ Experiences of Encountering Older People With Existential Loneliness, and Their Own Needs for Support

The study focusing on health care staff’s experience of encountering older people with EL [55] showed that HCPs perceived EL to appear in various forms associated with barriers in their encounters. The barriers described were as follows: (1) the older people’s bodily limitations (which complicated communication), demands, and needs perceived as insatiable by the staff, which, as a consequence, caused the staff to withdraw; (2) an older person’s need for personal privacy that was difficult to get through; or (3) fear and difficulty in encountering existential concerns on the behalf of health care staff [55].

#### Existential Loneliness in Relation to Different Care Contexts

So far, the impact of context has been analyzed in relation to the narratives from HCPs [56]. The results found differences and similarities among the care contexts concerning the professionals’ views on the origins of EL, the place of care, and the professionals’ own role. Concerning the origin of EL, the focus in home care and residential care was on life, the present and the past, compared with hospital and palliative care where the professionals mainly related EL to the forthcoming death. The older person’s home, as the place of care, was described to help to preserve the older person’s identity. In hospital and palliative care, as in institutional care, the place offered security, whereas in residential care, the place could make older people feel like strangers. Creating relationships was considered an important part of the professionals’ role in all 4 care contexts, although this had different meanings, purposes, and conditions [56]. This study will be completed with a reanalysis of the interviews with the older persons, sorted by context.

## Discussion

### Principal Findings

The results from the already published studies will now form the basis for the modeling process and the outcomes of the intervention. So far, we can conclude that an intervention targeting HCPs most certainly needs to be flexible and adjustable to different care contexts and designed as a pragmatic trial [36]. We have also learnt that the experience of EL is individual and that a person-centered approach is necessary. The intervention, thus, needs to include a toolbox of different approaches and methods.

During the progress of the studies performed so far, new areas of interest have emerged, such as contribution from volunteers, the perspective of managers in aged care settings, what kind of support significant others might need, and to what extent coordinators of support to relatives focus on EL in support groups. These studies are at present in progress and will also contribute to the overall design of the intervention program.

### Methodological Aspects

All qualitative research should be evaluated in terms of trustworthiness, including credibility, dependability, confirmability, and transferability [57]. One aspect of credibility is accuracy in the selection of informants. In the studies, we strove for a broad variation in experiences by involving older people representing a range of ages, civil statuses, phases of life, and care contexts. This also increases the transferability of the findings. To increase the credibility, we also strove for a variation concerning significant others: spouses, children, and other people close to the patient or resident. Concerning aspects of dependability, the same interview guide was used for all interviews with the same group of informants, and the process of data collection and analysis was closely monitored by the entire research group. The confirmability of the study concerns objectivity, accuracy of the material, relevance, and meaning [57]. In the interview studies, there was a designated group of senior and junior researchers and doctoral students involved in the data collection and analysis of each set of material. The entire research group will be involved in the synthesis of data, thereby enabling a multifaceted analysis while also reducing the impact of individual researchers’ preunderstanding of the phenomenon.

### Data Management and Quality Assurance

The confidentiality of the participants and the different care facilities involved has been maintained. Documentation of informed consent is kept in a safe, separate from the interview material, and it is thus impossible to link it to the interviews. Original recordings of the interviews are kept on a separate, protected hard drive. The transcripts have been coded, and names, places, and other information that might indicate or reveal the identity of the participants have been omitted. One of the junior researchers was assigned to act as a coordinator to collect and register all informed consent forms from the managers and the patients, and to act as a link between the researchers, the patients and residents, the significant others, and the HCPs. This coordinator was also responsible for storage of the informed consent documentation in a safe. All transcribed material has been organized and is kept in a separate safe. In seeking ethical approval for the study, we stated that the material will not be used for any other purposes and will therefore not be made available to any researchers outside the research group.

### The Research Group

The research group includes 5 senior and 3 junior researchers and 3 doctoral students, representing 3 universities in Southern Sweden. The research group also represents several areas, such as nursing, caring sciences, medical ethics, and human geography, thus increasing the possibility to illuminate the phenomenon and concept from different perspectives.

### Importance of the Study

The proportion of older people in the population is constantly increasing, and a growing number of older people will thus be the focus of care and services. The quality of care and services for older people is often debated in the media, usually from a perspective of misery. The present research project will contribute to knowledge that can strengthen HCPs and increase the well-being of patients and residents in the last phase of life. The studies are highly relevant from a human perspective in general, as death will inevitably strike us all, and for the situation of the HCPs in particular, as existential issues are one of the largest challenges in nursing care and care of older people. Knowledge and consciousness about different prerequisites for supporting patients, and their significant others’, experience of meaning at the end of life depending on the context of care provision also provides us with a basis for the development of an intervention directed toward increasing the ability of the HCPs to counter EL. This, in turn, will have a positive impact on the quality of care.

## Abbreviations

EL: existential loneliness
HCPs: health care professionals
MRC: Medical Research Council
